# Age-Specific Prostate Specific Antigen Cutoffs for Guiding Biopsy Decision in Chinese Population

**DOI:** 10.1371/journal.pone.0067585

**Published:** 2013-06-25

**Authors:** Rong Na, Yishuo Wu, Jianfeng Xu, Haowen Jiang, Qiang Ding

**Affiliations:** 1 Department of Urology, Huashan Hospital, Fudan University, Shanghai, China; 2 Center for Cancer Genomics, Wake Forest University School of Medicine, Winston-Salem, North Carolina, United States of America; 3 Urology Research Center, Fudan University, Shanghai, China; Innsbruck Medical University, Austria

## Abstract

**Background:**

Age-specific prostate specific antigen (PSA) cutoffs for prostate biopsy have been widely used in the USA and European countries. However, the application of age-specific PSA remains poorly understood in China.

**Methods:**

Between 2003 and 2012, 1,848 men over the age of 40, underwent prostate biopsy for prostate cancer (PCa) at Huashan Hospital, Shanghai, China. Clinical information and blood samples were collected prior to biopsy for each patient. Men were divided into three age groups (≤60, 61 to 80, and >80) for analyses. Digital rectal examination (DRE), transrectal ultrasound (prostate volume and nodule), total PSA (tPSA), and free PSA (fPSA) were also included in the analyses. Logistic regression was used to build the multi-variate model.

**Results:**

Serum tPSA levels were age-dependent (P = 0.008), while %fPSA (P = 0.051) and PSAD (P = 0.284) were age-independent. At a specificity of 80%, the sensitivities for predicting PCa were 83%, 71% and 68% with tPSA cutoff values of 19.0 ng/mL (age≤60),21.0 ng/mL (age 61–80), and 23.0 ng/mL (age≥81). Also, sensitivities at the same tPSA levels were able to reach relatively high levels (70%–88%) for predicting high-grade PCa. Area (AUC) under the receive operating curves (ROCs) of tPSA, %fPSA, PSAD and multi-variate model were different in age groups. When predicting PCa, the AUC of tPSA, %fPSA, PSAD and multi-variate model were 0.90, 0.57, 0.93 and 0.87 respectively in men ≤60 yr; 0.82, 0.70, 0.88 and 0.86 respectively in men 61–80 yr; 0.79, 0.78, 0.87 and 0.88 respectively in men>80 yr. When predicting Gleason Score ≥7 or 8 PCa, there were no significant differences between AUCs of each variable.

**Conclusion:**

Age-specific PSA cutoff values for prostate biopsy should be considered in the Chinese population. Indications for prostate biopsies (tPSA, %fPSA and PSAD) should be considered based on age in the Chinese population.

## Introduction

Prostate cancer (PCa) is the most common cancer and a leading cause of death among men in the world, with an estimated incidence of 903,500 cases, causing 258,400 death every year. [1] By the year of 2030, an estimated 500,000 men will die of PCa. [2] The incidence of PCa in China is still very low; however, it has risen rapidly over the last decade. [3], [4].

Prostate specific antigen (PSA) is one of the most important biomarkers for detecting prostate cancer and guiding decisions to biopsy the prostate. In addition PSA was found to have ability for predicting overall cancer mortality rate, while the reason was unclear. [5] The baseline level of PSA could even predict the probability of having PCa in the future for those who did not have PCa yet. [6] These findings broke though the traditional understanding of PSA testing. However, after decades of using PSA test, limitations have been observed, for instance, PSA test has low specificity (which causes the over-diagnosis), has poor ability to distinguish indolent form aggressive cancers (which causes the over-treatment for indolent PCa). [7], [8] Several tools were added to increase the specificity of PSA test, such as free PSA (fPSA), ratio of free to total PSA (%fPSA), PSA density (PSAD), age-specific PSA cutoff values, PSA isoforms (eg. p2PSA), etc. These additional tools have been proved to increase the accuracy of PCa diagnosis in different populations. [9], [10], [11], [12].

Age-specific cutoff values are used to aid urologists in making better biopsy decisions thereby reducing the number of unnecessary biopsies for a large group of men. Since its introduction into clinical practice, a PSA cutoff level of 4.0 ng/mL has been used for prostate biopsy. [13] However, several studies in the US and Europe have suggested that using a cuttoff of 4.0 ng/ml is inappropriate. The relationship between PSA, age, prostate volume and other factors have been extensively assessed in those studies, and significant associations were found. [14], [15], [16], [17], [18] Currently, age-specific PSA reference ranges (0–2.5 ng/mL for men 40–49 years old, 0–3.5 ng/mL for men 50–59 years old, 0–4.5 ng/mL for men 60–69 years old, and 0–6.5 ng/mL for men 70–79 years old) for PSA are widely used in the USA and European countries. [19], [20] In China, although several studies reported a relationship between age and PSA level, they were based on a healthy male population without PCa. [21], [22] The method used in those studies to set 95% percentiles for PSA levels in the population as reference range is inappropriate. And this was not helpful for the determination of prostate biopsy.

The goal of this study was to evaluate the relationship between PSA and age based on a biopsy population in China.

## Materials and Methods

### A. Study Population and Sample Collection

A total of 1,848 men that underwent prostate biopsy for PCa from 2003 to 2012 at Huashan Hospital, Fudan University, Shanghai, China, were included in the study. Huashan Hospital is one of the leading tertiary health institutes in China; and patients from all over the country seek their services. The characteristics of tertiary health institutes in China were described in our previous study. [23].

All men underwent ultrasound-guided transperineal needle prostate biopsy with 6 cores before Oct. 2007 or 10 cores thereafter. The indications for prostate biopsy at our institute were: (1) total PSA (tPSA)>4.0 ng/mL; (2) tPSA<4.0 ng/mL, with suspicious percentage of free PSA (%fPSA = fPSA/tPSA×100) (<0.16) or PSA density (PSAD = tPSA/prostate volume) (>0.15); (3) positive findings from digital rectal exam (DRE), with any level of tPSA; and (4) positive findings from imaging techniques, such as transrectal ultrasound and magnetic resonance imaging (MRI), with any level of tPSA. All specimens were diagnosed by pathologists from the Department of Pathology at our institute. Blood samples were collected prior to biopsy and were measured by the Department of Clinical Laboratory for tPSA and fPSA (measured by FDA approved technology Hitachi E170 before 2010 and Roche Cobas E602 thereafter). DRE results, transrectal ultrasound results (prostate volume = height ×length×width×0.52, measured by Aloka-α10 before 2010 and Hitachi EUB-7500 thereafter) and other clinical information were also collected prior to biopsy. Detailed indications for prostate biopsy and the rules of sample testing were described in our previous study. [23] Patients were excluded from the study if tPSA or pathology results were missing. Written informed consent was obtained from each patient for their participation. The study was approved by the Institutional Review Board of Huashan Hospital, Fudan University, Shanghai, China.

### B. Statistic Analysis

The men included in our study were divided into three age groups (≤60 yrs old, 61–80 yrs old and ≥81 yrs old). ANOVA tests were performed to compare the mean values of each variable for each group. Cross-tables were used to calculate sensitivities and specificities for detecting PCa vs. non-PCa in different age groups at the PSA cutoff level of 2.0, 3.0, 4.0, 6.0, 8.0, 10.0, 12.0, 15.0, 20.0, 25.0, 30.0, 35.0 ng/mL. The same method was used to calculate sensitivities and specificities for PSA and the detection of high grade prostate cancer (Gleason Score≥7 vs. Gleason Score<7 plus non-PCa; Gleason Score≥8 vs. Gleason Score<8 plus non-PCa) in different age groups at different PSA cutoff values. The area under a receiver operating characteristic (ROC) curve (AUC) of tPSA, %fPSA, PSAD and multi-variate model (analyzed by using multi-variate logistic regression including tPSA, %fPSA and total prostate volume) were measured within different age groups. Statistical analyses were performed using SPSS 19.0 (Statistical Product and Service Solutions, IBM Corporation, Armonk, New York, USA).

## Results

A total of 1,841 men were included in our study. Seven men were excluded because of missing information (i.e., tPSA or pathology results). Population characteristics and baseline information are shown in **Table 1**. There were 228 men (12.4%) under the age of 60, 1,365 men (74.1%) between 61 to 80 years old, and 248 men (13.5%) over the age of 80. Mean tPSA values of eachage group were significantly different (P = 0.008). Older men had higher tPSA levels. No significant differences were found in %fPSA (P = 0.051) and PSAD (P = 0.284) in age groups. A total of 857 out of 1,848 men (47%) were diagnosed with PCa. Thirty-four percent of men under the age of 60 had PCa compared with 46% in men aged 61 to 80 and 64% in men over the age of 80. Significant differences were observed among the groups (P = 1.44×10^−10^), for older men had higher percentage of PCa. Older men were significantly more likely to have Gleason scores ≥7 or Gleason score ≥8 (P = 2.62×10^−7^ and 0.005, respectively). The difference between detection rates based on 6-core biopsy and 10-core biopsy was not significant (Chi-square test, P = 0.976), therefore the cores of biopsies were not included in our analysis.

**Table 1:** Characteristics of the study population.

	Age				P†
	Age≤60 yr	Age 61–80 yr	Age≥81 yr	Total	
**Overall**	228 (12.4%)	1365 (74.1%)	248 (13.5%)	1841	-
tPSA (Mean±SD, ng/mL)*	19.0±4.0	21.7±3.4	27.2±3.7	22.0±3.5	0.008
%fPSA (Mean±SD)	14.4±0.1	16.8±0.1	17.2±0.1	16.6±0.1	0.051
PSAD (Mean±SD)	1.8±6.9	1.3±4.3	1.7±4.2	1.5±4.7	0.284
**Case vs. Control**					
PCa	77 (34%)	622 (46%)	158 (64%)	857 (47%)	1.44×10^−10^ ‡
Non-PCa	151 (66%)	743 (54%)	90 (36%)	984 (53%)	–
**Grade of diseases 1**					
Gleason Score<7	22 (10%)	186 (14%)	42 (17%)	250 (14%)	–
Gleason Score≥7	55 (24%)	436 (32%)	116 (47%)	607 (33%)	2.62×10^−7^ ‡
Non-PCa	151 (66%)	743 (54%)	90 (36%)	984 (53%)	–
**Grade of diseases 2**					
Gleason Score<8	40 (18%)	406 (30%)	42 (17%)	488 (27%)	–
Gleason Score≥8	37 (16%)	216 (16%)	116 (47%)	369 (20%)	0.005‡
Non-PCa	151 (66%)	743 (54%)	90 (36%)	984 (53%)	–

* The the means and standard deviations of tPSA were calculated based on logarithm of the original data. The results were taken exponential.

† The P-values were calculated by using one-way ANOVA to test whether there is any significant difference between the age groups (except “Total”).

‡ The P-values were calculated on the comparison among different age groups for PCa vs. non-PCa, Gleason Score≥7 vs. Gleason Score<7+ non-PCa, and Gleason Score≥8 vs. Gleason Score<8+ non-PCa, respectively.

**Figure 1:**
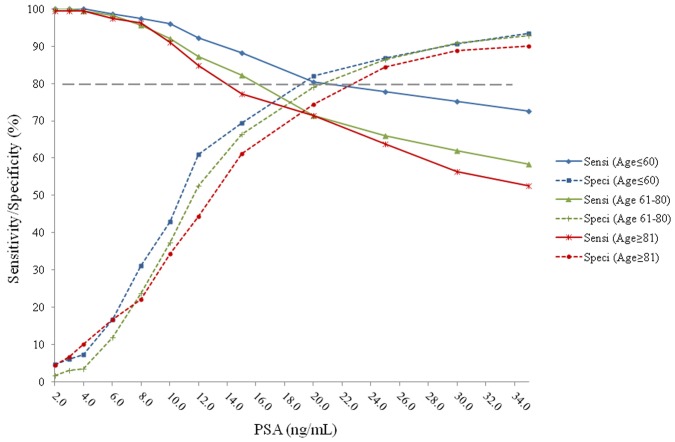
Sensitivities and specificities of PSA predicting PCa plus non-PCa in different age groups. When the specificities were 80%, the sensitivities were 83% with PSA value of 19.0 ng/mL in the age≤60 group; 71% with PSA value of 21.0 ng/mL in the age 61–80 group; and 68% with PSA value of 23.0 ng/mL in the age≥81 group.

**Figure 2:**
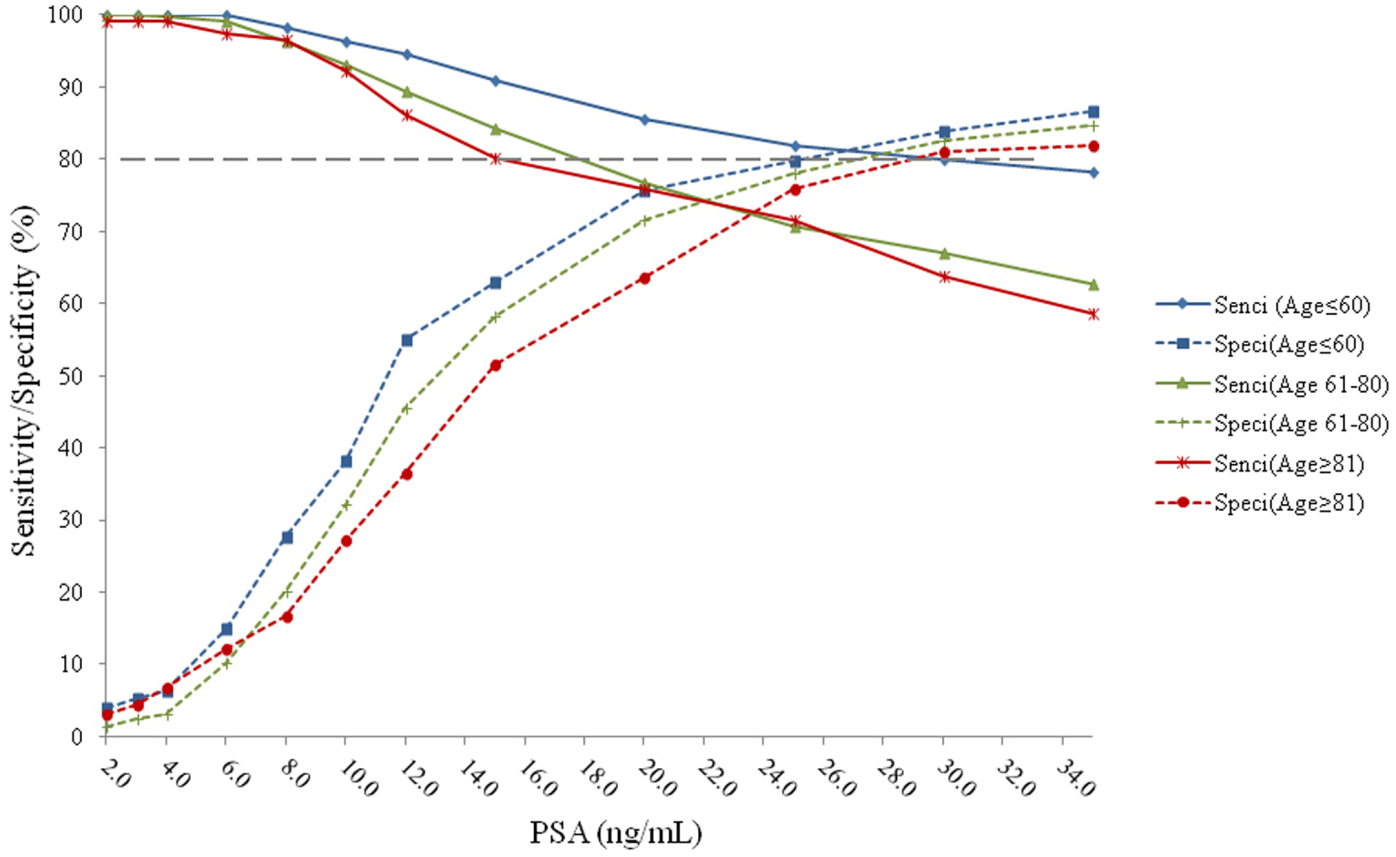
Sensitivities and specificities of PSA predicting Gleason Socre≥7 vs. Gleason Score <7 plus non-PCa in different age groups. The sensitivity was 86% and specificity was 73% with tPSA cutoff value of 19.0 ng/mL in the age≤60 group; the sensitivity was 75% and specificity was 72% with tPSA cutoff value of 21.0 ng/mL in the age 61–80 group; the sensitivity was 72% and specificity was 70% with PSA value of 23.0 ng/mL in the age≥81 group.

**Figure 3:**
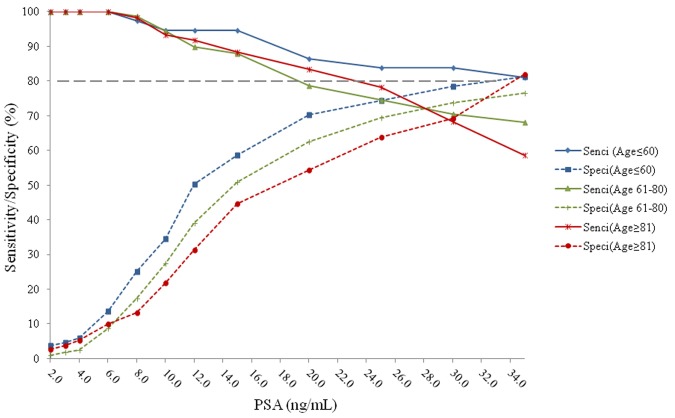
Sensitivities and specificities of PSA predicting Gleason Socre≥8 vs. Gleason Score <8 plus non-PCa in different age groups. The sensitivity was 88% and specificity was 69% with tPSA cutoff value of 19.0 ng/mL in the age≤60 group; the sensitivity was 78% and specificity was 62% with tPSA cutoff value of 21.0 ng/mL in the age 61–80 group; the sensitivity was 80% and specificity was 59% with PSA value of 23.0 ng/mL in the age≥81 group.

The sensitivities and specificities of PSA predicting PCa vs. non-PCa, Gleason Score≥7 PCa vs. Gleason Score<7 plus non-PCa, and Gleason Score≥8 PCa vs. Gleason Score<8 plus non-PCa are provided in **Figure 1** **,** **Figure 2** **, and** **Figure 3**. With a specificity of 80%, the sensitivities for predicting PCa vs. non-PCa were 83% with a tPSA cutoff value of 19.0 ng/mL in men ≤60 years old; 71% with tPSA cutoff value of 21.0 ng/mL in men 61 to 80 years old; and 68% with PSA value of 23.0 ng/mL in men ≥81 years old **(** **Figure 1** **)**. The cutoff value of 19.0 ng/mL in men ≤60 years old, 21.0 ng/mL in men 61 to 80 years old, and 23.0 ng/mL in men >80 years old, were then used to predict high grade and low grade PCa plus non-PCa. When predicting Gleason Score ≥7 PCa vs. Gleason Score<7 plus non-PCa, the sensitivity was 86% and specificity was 73% with tPSA cutoff value of 19.0 ng/mL in men ≤60 years old; the sensitivity was 75% and specificity was 72% with tPSA cutoff value of 21.0 ng/mL in men 61 to 80 years olf; the sensitivity was 72% and specificity was 70% with PSA value of 23.0 ng/mL in men ≥81 years old (**Figure 2**). When predicting Gleason Score ≥8 PCa vs. Gleason Score <8 plus non-PCa, the sensitivity was 88% and specificity was 69% with tPSA cutoff value of 19.0 ng/mL in men ≤60 years old; the sensitivity was 78% and specificity was 62% with tPSA cutoff value of 21.0 ng/mL in men 61 to 80 years old; the sensitivity was 80% and specificity was 59% with PSA value of 23.0 ng/mL in men ≥81 years old (**Figure 3**).

**Figure 4:**
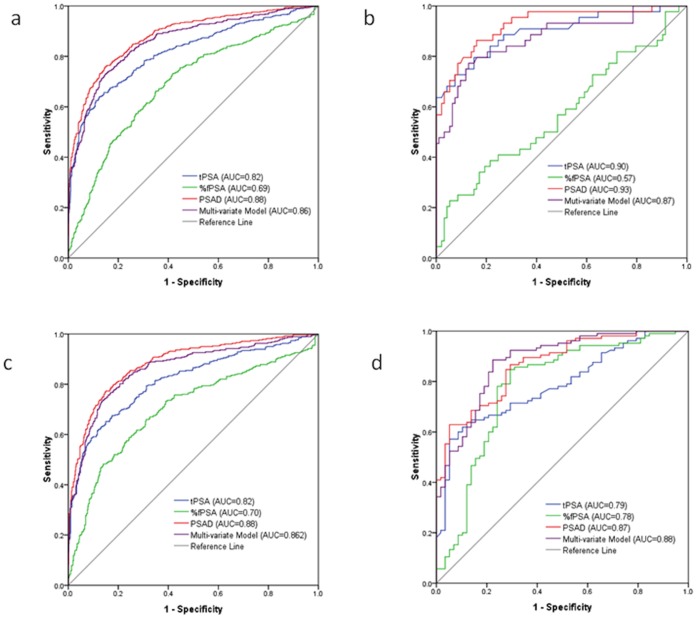
ROC of tPSA, %fPSA, PSAD and multi-variate model (including tPSA, %fPSA and prostate volume) for predicting the result of prostate biopsy for PCa vs. non-PCA in different age groups. (a)All study population: AUC-tPSA vs. AUC-%fPSA (P<0.001), AUC-PSAD vs. AUC-tPSA(P<0.001), AUC-model vs. AUC-tPSA(P<0.001); (b) Age≤60 yr, AUC-tPSA vs. AUC-%fPSA (P<0.001), AUC-PSAD vs. AUC-tPSA(P>0.05), AUC-model vs. AUC-tPSA(P>0.05); (c) Age 61–80 yr, AUC-tPSA vs. AUC-%fPSA (P<0.001), AUC-PSAD vs. AUC-tPSA(P = 0.001), AUC-model vs. AUC-tPSA(P = 0.02); (d) Age≥81 yr, AUC-tPSA vs. AUC-%fPSA (P>0.05), AUC-PSAD vs. AUC-tPSA(P>0.05), AUC-model vs. AUC-tPSA(P = 0.03).

**Figure 5:**
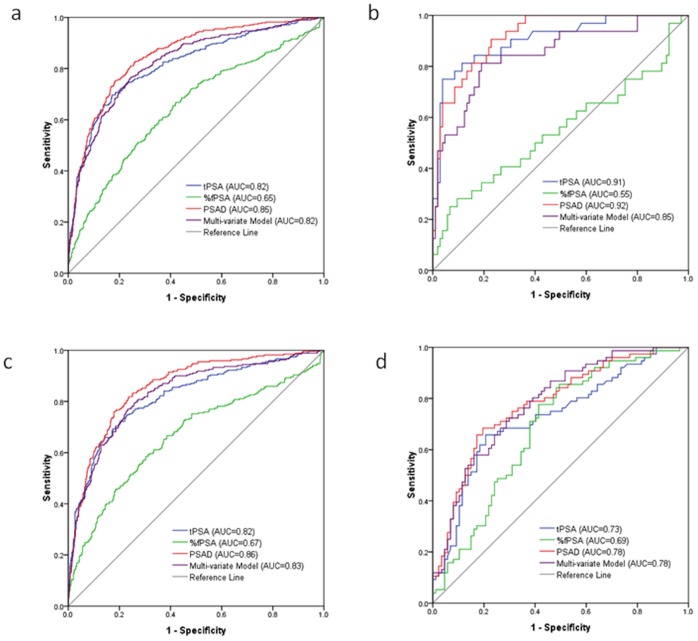
ROC of tPSA, %fPSA and PSAD for predicting the result of prostate biopsy for Gleason Score≥7 PCa vs. Gleason Score <7 and non-PCA in different age groups. (a)All study population: AUC-tPSA vs. AUC-%fPSA (P<0.001), AUC-PSAD vs. AUC-tPSA(P = 0.05), AUC-model vs. AUC-tPSA(P>0.05); (b) Age≤60 yr, AUC-tPSA vs. AUC-%fPSA (P>0.05), AUC-PSAD vs. AUC-tPSA(P>0.05), AUC-model vs. AUC-tPSA(P>0.05); (c) Age 61–80 yr, AUC-tPSA vs. AUC-%fPSA (P<0.001), AUC-PSAD vs. AUC-tPSA(P>0.05), AUC-model vs. AUC-tPSA(P>0.05); (d) Age≥81 yr, AUC-tPSA vs. AUC-%fPSA (P>0.05), AUC-PSAD vs. AUC-tPSA(P>0.05), AUC-model vs. AUC-tPSA(P>0.05).

**Figure 6:**
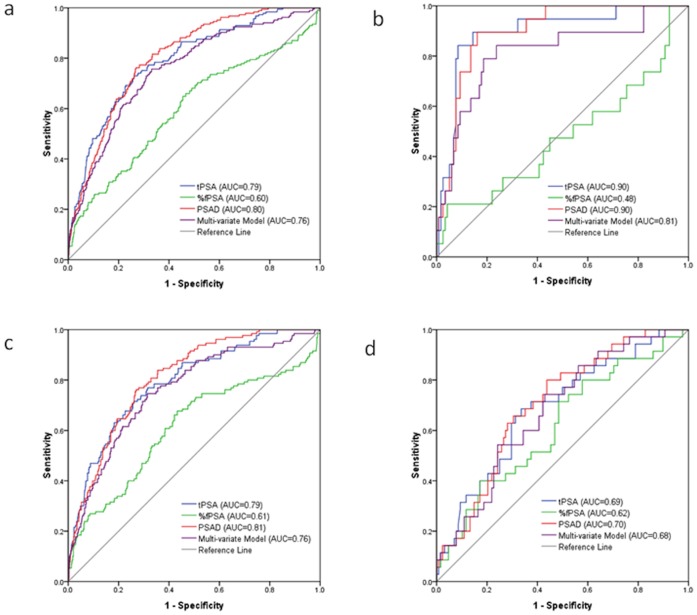
ROC of tPSA, %fPSA and PSAD for predicting the result of prostate biopsy for Gleason Score≥8 PCa vs. Gleason Score <8 and non-PCA in different age groups. (a)All study population: AUC-tPSA vs. AUC-%fPSA (P<0.001), AUC-PSAD vs. AUC-tPSA(P>0.05), AUC-model vs. AUC-tPSA(P>0.05); (b) Age≤60 yr, AUC-tPSA vs. AUC-%fPSA (P<0.001), AUC-PSAD vs. AUC-tPSA(P>0.05), AUC-model vs. AUC-tPSA(P<0.001); (c) Age 61–80 yr, AUC-tPSA vs. AUC-%fPSA (P<0.001), AUC-PSAD vs. AUC-tPSA(P>0.05), AUC-model vs. AUC-tPSA(P>0.05); (d) Age≥81 yr, AUC-tPSA vs. AUC-%fPSA (P>0.05), AUC-PSAD vs. AUC-tPSA(P>0.05), AUC-model vs. AUC-tPSA(P>0.05).

The ROC curves of PSA, %fPSA, PSAD and multi-variate model for predicting PCa vs. non-PCa, Gleason Score≥7 PCa vs. Gleason Score<7 plus non-PCa, and Gleason Score≥8 PCa vs. Gleason Score<8 plus non-PCa were presented in **Figure 4** **,** **Figure 5** **, and** **Figure 6** **, respectively**. Men with missing data (n = 630) were excluded from ROC analysis. When predicting PCa vs. non-PCa in the whole study population, the AUCs of PSAD and multi-variate model were 0.88 and 0.86, which was significantly higher than the AUCs of tPSA (AUC = 0.82, all P<0.010) and %fPSA (AUC = 0.69, P<0.001). The AUC of tPSA was significantly higher than the AUC of %fPSA (P<0.01) (**Figure 4** **a**). When predicting PCa vs. non-PCa in men ≤60 years old, there was no significant difference between the AUC of PSAD (AUC = 0.93) (or AUC of multi-variate model, AUC = 0.87) and the AUC of tPSA (AUC = 0.90), however, their AUCs were higher than the AUC of %fPSA (AUC = 0.57) (**Figure 4** **b**). Significant differences were observed between tests (P<0.01) when predicting PCa vs. non-PCa in men 61 to 80 years old (Figure 4c). Significant difference was observed between multi-varitate model (AUC = 0.88) and tPSA (AUC = 0.79, P = 0.03) (or %fPSA, P = 0.02) when predicting PCa vs. non-PCa in men ≥81 years old (**Figure 4d**). No significant differences between tPSA and PSAD (or multi-variate model) were found in any of the age groups (P>0.05) when predicting Gleason Score ≥7 PCa vs. Gleason Score <7 plus non-PCa or Gleason Score ≥8 PCa vs. Gleason Score <8 plus non-PCa (**Figure 5** **and** **Figure 6**). In addition, tPSA, PSAD, %fPSA and multi-variate model performed equally in the group of men ≥81 years old when predicting Gleason Score ≥7 PCa vs. Gleason Score <7 plus non-PCa or Gleason Score ≥8 PCa vs. Gleason Score<8 plus non-PCa (P>0.05) (**Figure 5** **and** **Figure 6**).

## Discussion

To the best of our knowledge, this is the first study to observe the relationship between PSA and age based in a Chinese prostate biopsy population. The relationship between AUCs (tPSA, PSAD, %fPSA and multi-variate model) and age were also observed in our study.

The characteristics of the study population showed that tPSA level was significantly related to age however, %fPSA and PSAD levels were not age dependent. These results were observed in other studies that were based on Caucasian populations. [14], [15], [16], [17], [18], [24], [25] We also observed that older men were at increased risk for PCa and high grade PCa (with a Gleason score ≥7 or 8). Most studies support the notion that tPSA levels increase with age which increases the risk of having PCa and high grade PCa. Currently, age-specific PSA reference ranges used in the USA and European countries are 0–2.5 ng/mL for men 40–49 years old, 0–3.5 ng/mL for men 50–59 years old, 0–4.5 ng/mL for men 60–69 years old, and 0–6.5 ng/mL for men 70–79 years old. In our study, we stratified the study population with the age range of ≤60 yrs old, 61–80 yrs old and ≥81 yrs old. This was because that we did not observe a significant difference of sensitivities and specificities between 61–70 year-old group and 71–80 year-old group in the current study.

Several studies have evaluated the use of reference ranges in Chinese men to determine the cutoff values of prostate biopsy decision. [21], [22] Those studies included large numbers of men without PCa.They evaluated tPSA levels and the upper 95th percentiles were considered as cutoff values for making prostate biopsy decisions. These cutoff values were chosen without the consideration of sensitivities and specificities in a non-case population, which were not suitable for making biopsy decision.

In a diagnostic study, Youden’s Index (Sensitivity+Specificity-1) was used to determine the cutoff value of diagnosis. Briefly, Youden’s Index values are larger when both sensitivity and specificity are higher, which indicate that the best cutoff has been identified. [26] However, we didn’t evaluate cutoff values for prostate biopsy decision using Youden’s Index in our study. The reason was that to choose the cutoff value for predicting PCa vs. non-PCa using Youden’s Index, we got cutoff values with higher specificities and lower sensitivities. For example, at the cutoff tPSA value of 25.0 ng/mL, we observed the highest Youden’s Index. And in this cutoff level, the sensitivity was 66.7% and specificity was 86.2% in the entire study population (not presented in this paper). Although, with tPSA testing, it is understandable to choose a cutoff value with higher specificity (fewer negative cases would undertake unnecessary prostate biopsy), the relatively higher specificities might cause a higher false negative (missing the PCa patients). We comprehensively evaluated the sensitivities and specificities in different situations. Firstly, based on a former study in our institute, the specificities ranked from 4.4% to 37.3% with tPSA values ranging 4–10 ng/mL. If the cutoff values were between 4–10 ng/mL, there might be large quantities of people who would undergo unnecessary biopsies. [23] On the other hand, it is necessary to have a cutoff value with relatively high sensitivity to predict high-grade PCa, to include more true positive cases (those who had high-grade PCa) and to lower the possibility of missing patients with high grade PCa. To balance the pros and cons, our aim was to find cutoff values in different age groups with higher specificities when predicting PCa and with higher sensitivities when predicting high-grade PCa. Therefore, we used a tPSA level with 80% specificity to predict PCa vs. non-PCa firstly, and then evaluated the sensitivities for predicting high-grade PCa (Gleason Score ≥7 or 8) with tPSA cutoff values in different age groups. All of the sensitivities at those tPSA levels (19.0 ng/mL in men ≤60 years old, 21.0 ng/mL in men 61 to 80 yeasr old, and 23.0 ng/mL in men ≥81 years old) were able to reach relatively high levels (70%–88%), which meant at those cutoff values, the majority of PCa cases or high-grade PCa cases could be detected. In addition, it is necessary to find the patients with younger age (and longer life expectancy) to provide curative, and to avoid overtreatment in the non-aggressive PCa patients with older age (shorter life expectancy). Thus, it is also understandable to use a relatively higher sensitivity cutoff value in younger men than in older men. In the current study, at the 90%sensitivity, the specificity for predicting PCa in the men ≤60 years old was 68% with the cutoff value of 14.0 ng/mL. At this cutoff value, the sensitivities for predicting Gleason Score ≥7 or 8 PCa were also >90%, while the specificities were around 55.0% to 60.0%. By using age-specific cutoff value of tPSA for prostate biopsy instead of using the cut of value of 4.0 ng/mL, we at least avoid 60–70% unnecessary prostate biopsy in men with elevated tPSA. Whether and how much the decision of the cutoff values may benefit the patients should be comprehensively evaluated in a systematic and prospective study with larger population in the future.

Compared with the other studies that were based on Caucasian men, our cutoff values were much higher. The American Urological Association recommends using the following cutoff values: 0–2.5 ng/mL for men 40–49 years old, 0–3.5 ng/mL for men 50–59 years old, 0–4.5 ng/mL for men 60–69 years old, and 0–6.5 ng/mL for men 70–79 years old. Studies have reported that at a cutoff level of 4.0 ng/mL, with a 6.2% false-positive rate (low overtreatment rate), only 20.5% of PCa cases could be detected (sensitivity). [27] Other studies based on Caucasian men also got the same results. [28], [29] We were able to detect 99.5% of PCa in our study, using a cutoff value of 4.0 ng/mL; however, 95.4% negative cases who would undertake unnecessary biopsies. Therefore, the cutoff values that we observed were considered appropriate in the study population and should be considered in a large population.

In this study, the AUCs of tPSA, %fPSA, PSAD and multi-vairate model (including tPSA, % fPSA and prostate volume) were also analyzed. Different performances were observed in different age groups. Especially, the multi-variate model (based on logistic regression) outperformed tPSA for predicting PCa, but did not have significant differences when predicting Gleason Score ≥7 or 8 PCa. This suggested that it might be useful to take these variates into consideration when making the decision of biopsy. The multi-variate model with new biomarkers (eg. p2PSA, PCA3, fusion genes) might be more accurate for predicting PCa or high grade PCa, however, were not able to evaluate in the current study. These new technologies were still unavailable in China. Thus, in different age groups, the value of these tests should be comprehensively evaluated in the clinical setting. These findings might be considered for prostate biopsy decision or diagnosis in the future.

One limitation of our study is that it is a retrospective study from only one health institute. However, as one of the tertiary health institutes in China, patients from all over the country come to our department; our study population could partially represent Chinese population. In addition, our study provided a good description of the relationship between age, tPSA, %fPSA, and PSAD, which might be useful for making prostate biopsy decisions in China in the future.

### Conclusions

Age-specific PSA cutoff values for guiding prostate biopsy decisions should be considered in the Chinese population. In addition, the value of tPSA, %fPSA and PSAD for prostate biopsies or PCa diagnosis should be differentiated based on age.
